# P-1251. Pharmacometric Analyses to Support Dose Selection of Zoliflodacin, a First-in-Class Oral Antibiotic Being Developed for the Treatment of Uncomplicated Gonorrhea

**DOI:** 10.1093/ofid/ofae631.1433

**Published:** 2025-01-29

**Authors:** Sujata M Bhavnani, Anthony P Cammarata, Jeffrey P Hammel, Alison Luckey, Pierre Daram, Kajal B Larson, John P O’Donnell, Christopher M Rubino

**Affiliations:** Institute for Clinical Pharmacodynamics, Schenectady, New York; Institute for Clinical Pharmacodynamics, Schenectady, New York; Institute for Clinical Pharmacodynamics, Schenectady, New York; Global Antibiotic R&D Partnership (GARDP), Geneva, Geneve, Switzerland; Global Antibiotic R&D Partnership (GARDP), Geneva, Geneve, Switzerland; Entasis Therapeutics Inc., an affiliate of Innoviva Specialty Therapeutics, Inc., Waltham, Massachusetts; Entasis Therapeutics Inc., an affiliate of Innoviva Specialty Therapeutics, Inc., Waltham, Massachusetts; Institute for Clinical Pharmacodynamics, Schenectady, New York

## Abstract

**Background:**

Zoliflodacin is a first-in-class oral bacterial gyrase inhibitor being developed as single dose treatment for uncomplicated gonorrhea. Population pharmacokinetic (PPK) analyses using data from 6 Phase 1 studies and the pivotal Phase 3 trial and assessments of probabilities of pharmacokinetic-pharmacodynamic (PK-PD) target attainment (PTA) were conducted to support zoliflodacin dose selection.

**Figure:**
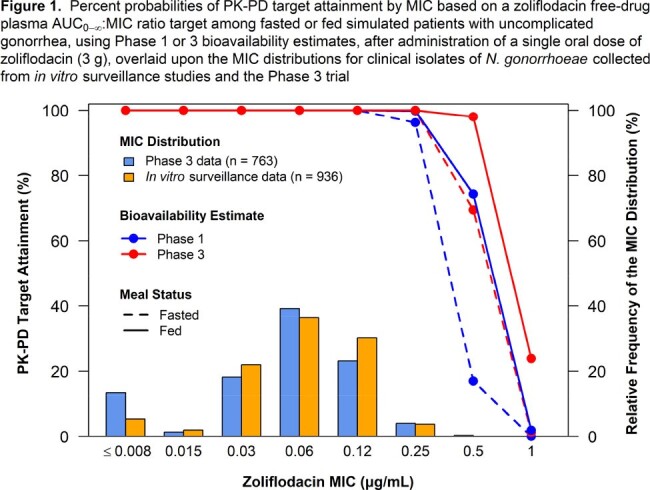

**Methods:**

PPK analyses were performed using plasma PK data from 261 participants, including 24 patients from the PK substudy of the pivotal Phase 3 trial. The final, qualified PPK model was used with non-clinical PK-PD and MIC data to perform PTA analyses among fed and fasted simulated patients with Phase 1 and 3 bioavailability estimates. The PK-PD index associated with efficacy is the ratio of free-drug plasma area under the curve from time zero to infinity (AUC_0-∞_) to MIC, with a target of 70.6 associated with suppression of *Neisseria gonorrhoeae* mutant amplification [Jacobsson *et al*., Front Pharmacol. 2021;12:682135]. PTA by MIC and averaged over the MIC distributions for Phase 3 and *in vitro* surveillance data was assessed among simulated patients.

**Results:**

A final PPK model with an oral depot compartment and associated relative bioavailability, a transit chain compartment to describe the absorption profile, and one systemic compartment with linear elimination best described the data. Body weight, pre-dose meal type, study phase, sex, formulation type or manufacturer, and concomitant administration of a strong CYP3A4 inhibitor were statistically significant predictors of inter-individual variability in zoliflodacin PK. Age and race were not identified as statistically significant covariates. PTA was ≥ 96.2% at a MIC ≤ 0.25 µg/mL (at which 100 and 99.6% of *in vitro* surveillance and Phase 3 isolates were inhibited) in simulated patients under fed and fasted conditions, and by bioavailability estimate/study phase (Figure 1). Averaged over either MIC distribution, PTA was ≥ 99.5% under all conditions assessed.

**Conclusion:**

A PPK model utilizing Phase 1 and 3 data described the zoliflodacin PK well. The high PTA (≥ 96.2 %) at a MIC ≤ 0.25 µg/mL and over the MIC distributions supported a 3 g single oral dose of zoliflodacin for the treatment of uncomplicated gonorrhea.

**Disclosures:**

**Sujata M. Bhavnani, PharmD; MS; FIDSA**, Achaogen Inc.: Grant/Research Support|Adagio Therapeutics, Inc.: Grant/Research Support|AiCuris Anti-infective Cures AG: Grant/Research Support|Albany Medical College: Grant/Research Support|AN2 Therapeutics: Grant/Research Support|Antabio SAS: Grant/Research Support|Apogee Biologics, Inc.: Grant/Research Support|Arcutis Biotherapeutics, Inc.: Grant/Research Support|B. Braun Medical Inc.: Grant/Research Support|Basilea Pharmaceutica: Grant/Research Support|BioFire Diagnostics, LLC.: Grant/Research Support|Cidara Therapeutics Inc.: Grant/Research Support|Cipla USA: Grant/Research Support|Cumberland Pharmaceuticals Inc.: Grant/Research Support|Entasis Therapeutics Inc., an affiliate of Innoviva Specialty Therapeutics, Inc.: Grant/Research Support|Excalibur Pharmaceuticals Inc.: Grant/Research Support|Fedora Pharmaceuticals: Grant/Research Support|Genetech: Grant/Research Support|GlaxoSmithKline: Advisor/Consultant|GlaxoSmithKline: Grant/Research Support|Global Antibiotic Research and Development Partnership: Grant/Research Support|Hoffmann-La Roche: Grant/Research Support|Inoterm: Grant/Research Support|Insmed Inc.: Grant/Research Support|Institute for Clinical Pharmacodynamics, Inc.: Ownership Interest|Iterum Therapeutics Limited: Grant/Research Support|Kaizen Bioscience: Grant/Research Support|Lassen Therapeutics Inc.: Grant/Research Support|Matinas Biopharma: Grant/Research Support|Meiji Seika Pharma Co., Ltd.: Grant/Research Support|Melinta Therapeutics: Grant/Research Support|Mutabilis: Grant/Research Support|Nabriva Therapeutics AG: Grant/Research Support|Novobiotic Pharmaceuticals LLC.: Grant/Research Support|Paratek Pharmaceuticals, Inc.: Grant/Research Support|Pfizer Inc.: Grant/Research Support|Praxis Precision Medicines, Inc.: Grant/Research Support|PTC Therapeutics: Grant/Research Support|PureTech LYT 100 Inc.: Grant/Research Support|Qpex Biopharma: Grant/Research Support|Renibus Therapeutics: Grant/Research Support|Sfunga Therapeutics: Grant/Research Support|Shionogi Inc.: Advisor/Consultant|Shionogi Inc.: Grant/Research Support|Spero Therapeutics: Grant/Research Support|Spruce Biosciences Inc.: Grant/Research Support|Suzhou Sinovent Pharmaceuticals Co.: Grant/Research Support|Theravance: Grant/Research Support|University of Wisconsin: Grant/Research Support|US Food and Drug Administration: Grant/Research Support|UT Southwestern: Grant/Research Support|ValanBio Therapeutics, Inc.: Grant/Research Support|VenatoRx: Grant/Research Support|Zogenix International: Grant/Research Support **Anthony P. Cammarata, M.S.**, Achaogen Inc.: Grant/Research Support|Adagio Therapeutics, Inc.: Grant/Research Support|AiCuris Anti-infective Cures AG: Grant/Research Support|Albany Medical College: Grant/Research Support|AN2 Therapeutics: Grant/Research Support|Antabio SAS: Grant/Research Support|Apogee Biologics, Inc.: Grant/Research Support|Arcutis Biotherapeutics, Inc.: Grant/Research Support|B. Braun Medical Inc.: Grant/Research Support|Basilea Pharmaceutica: Grant/Research Support|BioFire Diagnostics, LLC.: Grant/Research Support|Cidara Therapeutics Inc.: Grant/Research Support|Cipla USA: Grant/Research Support|Cumberland Pharmaceuticals Inc.: Grant/Research Support|Entasis Therapeutics Inc., an affiliate of Innoviva Specialty Therapeutics, Inc.: Grant/Research Support|Excalibur Pharmaceuticals Inc.: Grant/Research Support|Fedora Pharmaceuticals: Grant/Research Support|Genetech: Grant/Research Support|GlaxoSmithKline: Grant/Research Support|Global Antibiotic Research and Development Partnership: Grant/Research Support|Hoffmann-La Roche: Grant/Research Support|Inotrem: Grant/Research Support|Insmed Inc.: Grant/Research Support|Institute for Clinical Pharmacodynamics, Inc.: Employee|Iterum Therapeutics Limited: Grant/Research Support|Kaizen Bioscience: Grant/Research Support|Lassen Therapeutics Inc.: Grant/Research Support|Matinas Biopharma: Grant/Research Support|Meiji Seika Pharma Co., Ltd.: Grant/Research Support|Melinta Therapeutics: Grant/Research Support|Mutabilis: Grant/Research Support|Nabriva Therapeutics AG: Grant/Research Support|Novobiotic Pharmaceuticals LLC.: Grant/Research Support|Paratek Pharmaceuticals, Inc.: Grant/Research Support|Pfizer Inc.: Grant/Research Support|Praxis Precision Medicines, Inc.: Grant/Research Support|PTC Therapeutics: Grant/Research Support|PureTech LYT 100 Inc.: Grant/Research Support|Qpex Biopharma: Grant/Research Support|Renibus Therapeutics: Grant/Research Support|Sfunga Therapeutics: Grant/Research Support|Shionogi Inc.: Grant/Research Support|Spero Therapeutics: Grant/Research Support|Spruce Biosciences Inc.: Grant/Research Support|Suzhou Sinovent Pharmaceuticals Co.: Grant/Research Support|Theravance: Grant/Research Support|University of Wisconsin: Grant/Research Support|US Food and Drug Administration: Grant/Research Support|UT Southwestern: Grant/Research Support|ValanBio Therapeutics, Inc.: Grant/Research Support|VenatoRx: Grant/Research Support|Zogenix International: Grant/Research Support **Jeffrey P. Hammel, MS**, Achaogen Inc.: Grant/Research Support|Adagio Therapeutics, Inc.: Grant/Research Support|AiCuris Anti-infective Cures AG: Grant/Research Support|Albany Medical College: Grant/Research Support|AN2 Therapeutics: Grant/Research Support|Antabio SAS: Grant/Research Support|Apogee Biologics, Inc.: Grant/Research Support|Arcutis Biotherapeutics, Inc.: Grant/Research Support|B. Braun Medical Inc.: Grant/Research Support|Basilea Pharmaceutica: Grant/Research Support|BioFire Diagnostics, LLC.: Grant/Research Support|Cidara Therapeutics Inc.: Grant/Research Support|Cipla USA: Grant/Research Support|Cumberland Pharmaceuticals Inc.: Grant/Research Support|Entasis Therapeutics Inc., an affiliate of Innoviva Specialty Therapeutics, Inc.: Grant/Research Support|Excalibur Pharmaceuticals Inc.: Grant/Research Support|Fedora Pharmaceuticals: Grant/Research Support|Genetech: Grant/Research Support|GlaxoSmithKline: Grant/Research Support|Global Antibiotic Research and Development Partnership: Grant/Research Support|Hoffmann-La Roche: Grant/Research Support|Inotrem: Grant/Research Support|Insmed Inc.: Grant/Research Support|Institute for Clinical Pharmacodynamics, Inc.: Employee|Iterum Therapeutics Limited: Grant/Research Support|Kaizen Bioscience: Grant/Research Support|Lassen Therapeutics Inc.: Grant/Research Support|Matinas Biopharma: Grant/Research Support|Meiji Seika Pharma Co., Ltd.: Grant/Research Support|Melinta Therapeutics: Grant/Research Support|Mutabilis: Grant/Research Support|Nabriva Therapeutics AG: Grant/Research Support|Novobiotic Pharmaceuticals LLC.: Grant/Research Support|Paratek Pharmaceuticals, Inc.: Grant/Research Support|Pfizer Inc.: Grant/Research Support|Praxis Precision Medicines, Inc.: Grant/Research Support|PTC Therapeutics: Grant/Research Support|PureTech LYT 100 Inc.: Grant/Research Support|Qpex Biopharma: Grant/Research Support|Renibus Therapeutics: Grant/Research Support|Sfunga Therapeutics: Grant/Research Support|Shionogi Inc.: Grant/Research Support|Spero Therapeutics: Grant/Research Support|Spruce Biosciences Inc.: Grant/Research Support|Suzhou Sinovent Pharmaceuticals Co.: Grant/Research Support|Theravance: Grant/Research Support|University of Wisconsin: Grant/Research Support|US Food and Drug Administration: Grant/Research Support|UT Southwestern: Grant/Research Support|ValanBio Therapeutics, Inc.: Grant/Research Support|VenatoRx: Grant/Research Support|Zogenix International: Grant/Research Support **Alison Luckey, MD**, GARDP: Employee of GARDP, a non-profit Swiss-based foundation, which receives government and private-funded institutional grants/research support **Pierre Daram, Ph.D.**, GARDP: GARDP (Employee of GARDP, a non-profit Swiss-based foundation, which receives government and private-funded institutional grants/research support) **Kajal B. Larson, Ph.D.**, Entasis Therapeutics Inc., an affiliate of Innoviva Specialty Therapeutics, Inc.: Employee|Entasis Therapeutics Inc., an affiliate of Innoviva Specialty Therapeutics, Inc.: Stocks/Bonds (Public Company) **John P. O'Donnell, B.S.**, Entasis Therapeutics Inc., an affiliate of Innoviva Specialty Therapeutics, Inc.: Employee|Entasis Therapeutics Inc., an affiliate of Innoviva Specialty Therapeutics, Inc.: Stocks/Bonds (Public Company) **Christopher M. Rubino, PharmD**, Achaogen Inc.: Grant/Research Support|Adagio Therapeutics, Inc.: Grant/Research Support|AiCuris Anti-infective Cures AG: Grant/Research Support|Albany Medical College: Grant/Research Support|AN2 Therapeutics: Grant/Research Support|Antabio SAS: Grant/Research Support|Apogee Biologics, Inc.: Grant/Research Support|Arcutis Biotherapeutics, Inc.: Grant/Research Support|B. Braun Medical Inc.: Grant/Research Support|Basilea Pharmaceutica: Grant/Research Support|BioFire Diagnostics, LLC.: Grant/Research Support|Cidara Therapeutics Inc.: Grant/Research Support|Cipla USA: Grant/Research Support|Cumberland Pharmaceuticals Inc.: Grant/Research Support|Entasis Therapeutics Inc., an affiliate of Innoviva Specialty Therapeutics, Inc.: Grant/Research Support|Excalibur Pharmaceuticals Inc.: Grant/Research Support|Fedora Pharmaceuticals: Grant/Research Support|Genentech: Grant/Research Support|GlaxoSmithKline: Grant/Research Support|Global Antibiotic Research and Development Partnership: Grant/Research Support|Hoffmann-La Roche: Grant/Research Support|Inotrem: Grant/Research Support|Insmed Inc.: Grant/Research Support|Institute for Clinical Pharmacodynamics, Inc.: Ownership Interest|Iterum Therapeutics Limited: Grant/Research Support|Kaizen Bioscience: Grant/Research Support|Lassen Therapeutics Inc.: Grant/Research Support|Matinas Biopharma: Grant/Research Support|Meiji Seika Pharma Co., Ltd.: Grant/Research Support|Melinta Therapeutics: Grant/Research Support|Mutabilis: Grant/Research Support|Nabriva Therapeutics AG: Grant/Research Support|Novobiotic Pharmaceuticals LLC.: Grant/Research Support|Paratek Pharmaceuticals, Inc.: Grant/Research Support|Pfizer Inc.: Grant/Research Support|Praxis Precision Medicines, Inc.: Grant/Research Support|PTC Therapeutics: Grant/Research Support|PureTech LYT 100 Inc.: Grant/Research Support|Qpex Biopharma: Grant/Research Support|Renibus Therapeutics: Grant/Research Support|Sfunga Therapeutics: Grant/Research Support|Shionogi Inc.: Grant/Research Support|Spero Therapeutics: Grant/Research Support|Spruce Biosciences Inc.: Grant/Research Support|Suzhou Sinovent Pharmaceuticals Co.: Grant/Research Support|Theravance: Grant/Research Support|University of Wisconsin: Grant/Research Support|US Food and Drug Administration: Grant/Research Support|UT Southwestern: Grant/Research Support|ValanBio therapeutics, Inc.: Grant/Research Support|VenatoRx: Grant/Research Support|Zogenix International: Grant/Research Support

